# Performance-based criteria for safe and circular digestate use in agriculture

**DOI:** 10.1038/s41598-025-33314-x

**Published:** 2025-12-24

**Authors:** Thuane Mendes Anacleto, Helena Rodrigues Oliveira, Giacomo Carraro, Polina Skvortsova, Luka Šafarič, Sepehr Yekta Shakeri, Annika Björn, Érika Flávia Machado Pinheiros, Alex Enrich-Prast

**Affiliations:** 1 Programa de Pós-Graduação em Biotecnologia Vegetal e Bioprocessos, Universidade Federal do Rio de Janeiro, Rio de Janeiro, Brazil; 2 Unidade Multiusuário de Análises Ambientais, Instituto de Biologia, Universidade Federal do Rio de Janeiro, Rio de Janeiro, Brazil; 3 Biogas Solutions Research Center, Linköping University, Linköping, Sweden; 4 Centro Federal de Educação Tecnológica Celso Suckow da Fonseca (CEFET/RJ), Rio de Janeiro, Brazil; 5 Department of Thematic Studies – Environmental Change, Linköping University, Linköping, Sweden; 6 Department of Ecology and Environmental Protection Technologies, Sumy State University, Sumy, Ukraine; 7 Instituto de Agronomia, Universidade Federal Rural do Rio de Janeiro, Rio de Janeiro, Brazil; 8 Institute of Marine Science, Federal University of São Paulo (IMar/UNIFESP), Santos, Brazil

**Keywords:** Biofertilizer, Phytotoxicity, Circular economy, Nutrient recovery, Solid-liquid separation, Ecology, Ecology, Environmental sciences

## Abstract

Anaerobic digestion converts organic waste into renewable energy (biogas) and recyclable nutrients (digestate), generating over one billion tons of digestate annually. While this represents a major resource, its safe reuse remains a bottleneck for nutrient circularity, particularly for closing global nitrogen loops. We analyzed digestates from 23 full-scale digesters in Sweden, Norway, and Denmark across whole, liquid, and solid fractions using germination index (GI) assays and chemical profiling. Three parameters predicted phytotoxicity: total ammonia nitrogen (TAN ≥ 1,122 mg N L^− 1^), potassium (K ≥ 39.6 × 10^3^ mg kg^− 1^), and boron (B ≥ 22.5 mg kg^− 1^). When all thresholds were exceeded, germination indices dropped below 50% in every case. Based on these findings, we propose a decision-ready framework linking TAN-K-B thresholds to germination outcomes, guiding mitigation through acidification, stripping, blending, or source control. This outcome-based screening reduces monitoring complexity while maintaining compliance with EU and US pollutant ceilings. Its implementation strengthens nitrogen use efficiency, curbs NH_3_ and N_2_O emissions, and secures crop establishment. By shifting from origin-based restrictions to performance-based thresholds, our framework provides transparent certification, builds farmer confidence, and positions digestate reuse as a global lever for climate mitigation, nutrient circularity, and food system resilience.

**Supplementary Information:**

The online version contains supplementary material available at 10.1038/s41598-025-33314-x.

## Introduction

Closing nutrient loops is a key to the circular bioeconomy and a prerequisite for remaining within planetary boundaries for nitrogen (N) and phosphorus (P). However, current food and energy systems continue to drive boundary transgression, with cascading impacts on ecosystems and climate^1^. Anaerobic digestion (AD) is pivotal to this transition, simultaneously producing renewable energy and recycling nutrients from organic waste streams, thereby advancing net-zero carbon and climate targets for global energy transitions^2^. Estimates suggest > 132,000 industrial-scale plants and ~ 50 million micro-digesters operate worldwide, with capacity growing at ~ 13% annually^2–5^. As part of the EU’s REPowerEU plan, biogas infrastructure is being scaled dramatically, targeting 35 billion m^3^ of biomethane per year by 2030 and an outlook to 151 billion m^3^ by 2050^6^.

However, digestate generation currently exceeds one billion tons annually worldwide^2–5^. If not properly managed, it can lead to major N losses (up to 70% following direct land application through ammonia volatilization^7^, nutrient runoff, contaminant accumulation in soils, and carbon losses^8–10^. Rather than a liability, this volume can be an asset for N circularity. European strategies such as the Green Deal and the Farm to Fork plan target a 50% reduction in nutrient losses by 2030, emphasizing efficient N recycling and reduced reliance on Haber-Bosch fertilizers^4,11,12^. In this context, the “circular nitrogen economy” routes agricultural residues and waste-derived streams into safe fertilizer pathways, provided performance metrics guide deployment^13^.

When properly handled, digestate becomes a valuable economic asset, a biofertilizer that can displace fossil-derived synthetic fertilizers, providing up to ~ 78 kg N ha^− 1^ and ~ 33 kg P_2_O_5_ ha^− 1^ per application when optimized through acidification, injection, and solid-liquid separation^14^. Such substitution could offset a significant share of the greenhouse gas (GHG) emissions associated with synthetic N fertilizers. More precisely, the production of synthetic N fertilizers accounts for ~ 40% of their life-cycle emissions, equivalent to ~ 0.8% of the global GHG emissions^12,15^, alleviate pressure on finite phosphate reserves concentrated in few geopolitical regions^16^, and help realign global N and P cycles with planetary boundaries.

Agronomically, digestate can improve soil structure, N retention, crop yields, and stimulate soil microbial activity, including antibiotic-producing actinomycetes^8,17–19^. Despite this potential, current regulations often apply origin-based restrictions rather than performance-based criteria. For example, the EU Fertilizing Products Regulation (2019/1009), renders sewage sludge digestates ineligible for Conformité Européenne (CE) marking as fertilizing products, regardless of their quality^20^, while U.S. biosolids rules (EPA 40 CFR Part 503) apply pollutant limits, hygiene classes, and management standards. Similar regulatory frameworks exist in Brazil (CONAMA Resolution 498/2020)^21^, and China (GB 4282–2018; GB/T 24600–2009)^21,22^.

Despite these regulatory constraints, strategies that separate nutrient delivery from contaminant load offer a pathway to broaden digestate reuse while meeting safety standards. Solid-liquid separation, one of the most widely implemented approaches in full-scale biogas plants, partitions contaminants, such as heavy metals, concentrating them in the solid fraction and thereby facilitating compliance of the liquid fraction with regulatory thresholds^11,23^.

The germination index (GI) is a validated bioassay that integrates seed germination and root elongation, capturing both lethal and sublethal effects. Standardized thresholds – GI > 80% (non-phytotoxic), 50–80% (moderately phytotoxic), < 50% (highly phytotoxic) – enable policy-relevant assessment and complement chemical analyses in identifying hazards, such as heavy metals and pesticides, in line with established guidelines (OECD TG 208; ISO 11269-2)^24^ . GI tests are commonly performed using tomato seeds (*Solanum lycopersicum L.*), a sensitive species for herbicides and pollutants detection^25,26^. Even herbicides considered low-risk by the European Food Safety Authority (EFSA), such as aminopyralid, can reduce tomato yields by up to 95% at 0.2 µg kg^− 1^ in soil^26–28^.

Although several studies have reported stimulatory or phytotoxic digestate effects, outcomes are strongly dependent on origin, treatment, and dose. Seed bioassays often show stimulation at low application rates but inhibition at higher doses, with GI reductions closely linked to elevated total ammonia nitrogen (TAN) and salinity^14,29^. In soil incubations, free NH_3_ accumulation at alkaline pH impairs root elongation, while excessive K and B drive osmotic stress and micronutrient antagonisms^30^. Liquid digestates tends to be less inhibitory than solid digestate, which concentrate heavy metals and persistent organic contaminants^14,29^.

However, most of the current evidence comes from lab-scale or single-feedstock trials, which, while valuable to highlight key digestate properties, provide limited guidance for defining broadly applicable performance-based criteria^3,24,29,31^. To fill this gap, this study evaluated 23 full-scale digestates from sewage sludge, food waste, agricultural biomass, and manure, across solid (SD), liquid (LD), and whole (WD) digestate fractions. By combining GI bioassessment with detailed chemical profiling and statistical modeling we aim to identify robust, performance-based criteria for safe and circular agricultural use.

## Results

### Phytotoxicity variation with feedstock, fraction, and composition

**Fig. 1:**
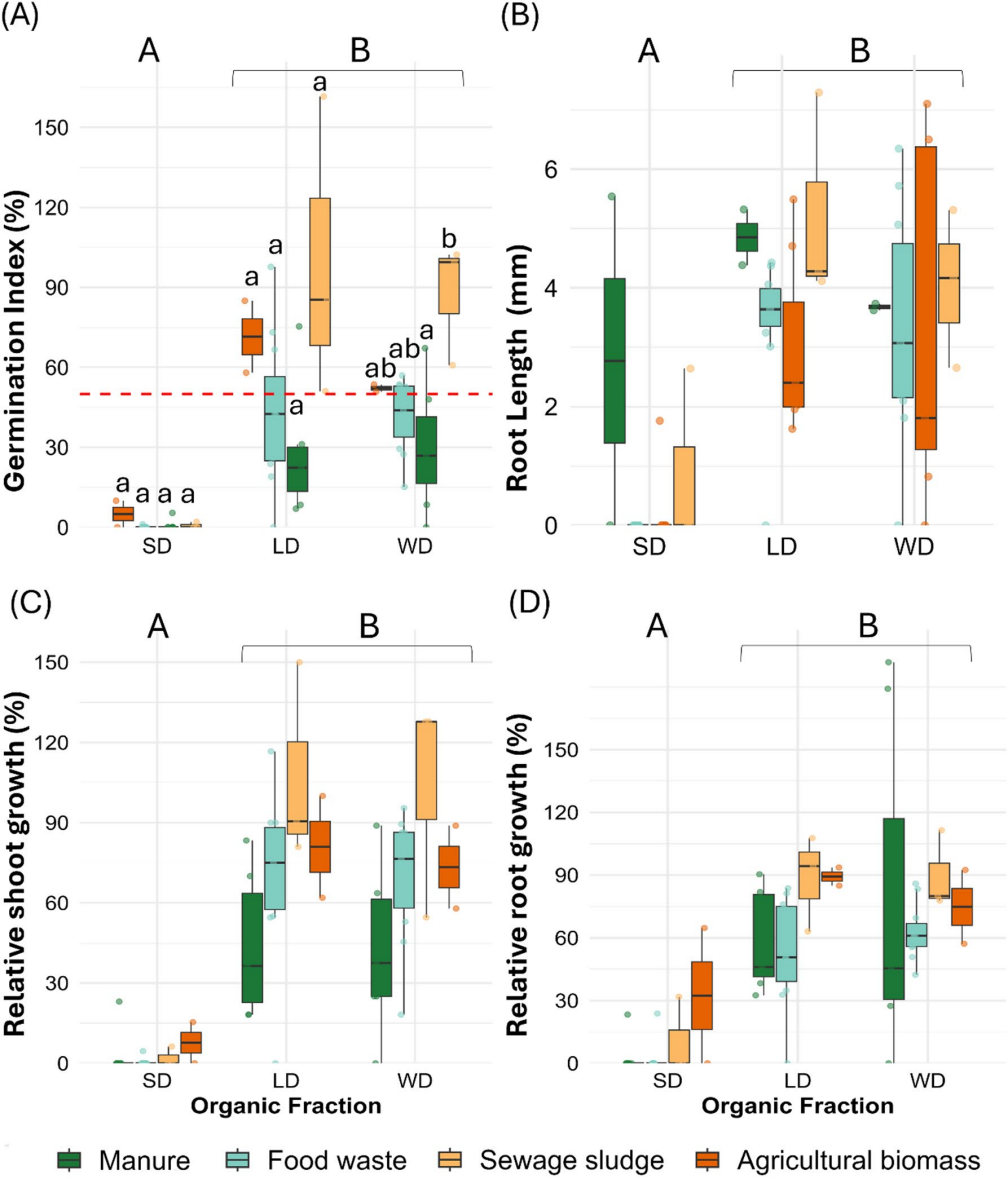
Effects of digestate fraction and feedstock origin on seed germination. Boxplots show medians (center line) and interquartile ranges (IQR; boxes, Q1–Q3); whiskers extend to 1.5×IQR, and points represent individual observations. (**A**) germination index (%), red dashed line represents the high-phytotoxic effect limit (50%), (**B**) Root length (mm), (**C**) Relative shoot growth (%), and (**D**) Relative root growth (%) of tomato seeds after 72 h exposure. Digestates from 23 full-scale digesters deriving from four main feedstocks: agricultural biomass (*n* = 2; orange), food waste (*n* = 11; blue), manure (*n* = 7; green), and sewage sludge (*n* = 3; yellow) were separated into three fractions: liquid (LD), solid (SD), and whole digestate (WD). Lowercase letters indicate difference among feedstocks within each fraction; uppercase letters indicate differences among fractions (Kruskal-Wallis test with Dunn’s test; Bonferroni-adjusted α = 0.05).

Digestates from 23 full-scale digesters showed wide variation across main feedstocks (sewage sludge, manure, food waste, agricultural biomass) and fractions (WD, LD, and SD). GI ranged from < 1% in SD of food waste and manure to > 99% in WD from sewage sludge (Fig. 1A; Supplementary Table 1). Sewage sludge digestates consistently exhibited the highest GI medians, coupled with robust root elongation in LD (4.3 mm) and balanced relative shoot and root growth ratios (RSG-WD: 127.8%; RRG-LD: 94.3%; Fig. 1B-D; Supplementary Table 1). Agricultural biomass digestates generally exceeded the 50% threshold commonly used to denote high phytotoxicity^23^, whereas manure and food waste digestates were uniformly phytotoxic, with GI medians, below thresholds for safe agronomic use.

Fractionation strongly modulated phytotoxicity. Sewage sludge WD (GI: 99%; IQR 80%–101%) and LD (GI: 85%; IQR 68%–123%) consistently outperformed SD (GI: 0%; IQR 0%–0.1%; Fig. 1A). Across feedstocks, SD showed near-zero GI in manure, food waste and sewage sludge (all < 1%; IQR 0%–0.1%), whereas agricultural biomass SD was the only non-zero case (GI: 5.0% IQR 2.5–7.5%). In contrast, LD and WD were substantially higher, e.g., food waste LD (GI: 42.5%; IQR 24.9–56.5%), WD (GI: 43.9%; IQR 33.8–52.9%), and agricultural biomass LD (GI: 71.5%; IQR 64.7–78.2%), WD (GI: 52.2%; IQR 51.5–52.8%; Supplementary Table 1). Patterns for root length, RSG and RRG were consistent with GI across fractions (Fig. 1B–D), and differences between LD and WD were generally small and feedstock-specific.

Solid-liquid separation concentrates particulate material in the solid fraction, resulting in a much higher TS content in SD, and likely leading to the accumulation of specific inhibitory compounds. Since the germination tests were performed on a fresh mass basis rather than normalized to TS content, the higher solids content of SD could potentially inflate the observed inhibitory response. However, applying digestate on a wet-mass basis reflects typical field practices, where application rates are determined by fresh mass or volume rather than dry-matter equivalence. We estimate that the overall toxicity pattern (SD > LD) would remain unchanged even if applications were normalized by TS or VS, as SD retains the largest proportion of low-solubility and recalcitrant inhibitory constituents, maintaining its high phytotoxic profile independent of the mass-loading bias.

### Chemical drivers of germination responses

**Fig. 2:**
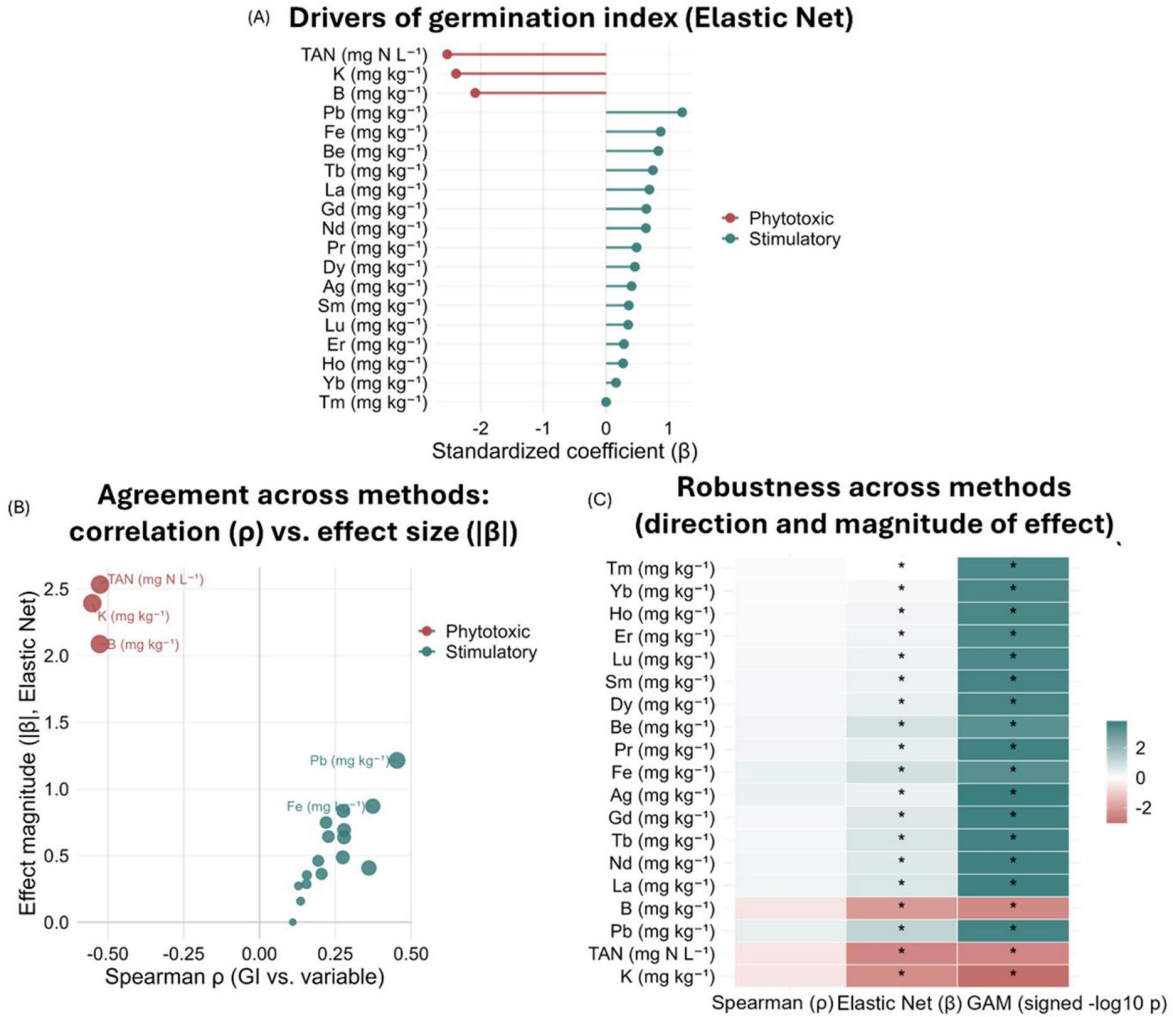
Independent chemical drivers of the germination index (GI) and agreement across methods. (**A**) Standardized elastic-net (EN) coefficients (β, standardized regression coefficients) for GI, indicating stimulatory (green) or phytotoxic (red) effects; (**B**) Agreement between bivariate and multivariate signals: Spearman’s rank correlation coefficient (ρ) versus |β|; point size encodes, log_10_ (FDR), the negative base-10 logarithm of the false discovery rate (FDR), adjusted p-value; (**C**) Robustness across methods: heatmap of signed effects from Spearman (ρ), EN (β), and generalized additive models (GAMs), shown as signed − log_10_ p; * denote significance (FDR < 0.05 for Spearman; β ≠ 0 for EN; *p* < 0.05 for GAM). TAN: total ammonia nitrogen.

**Fig. 3:**
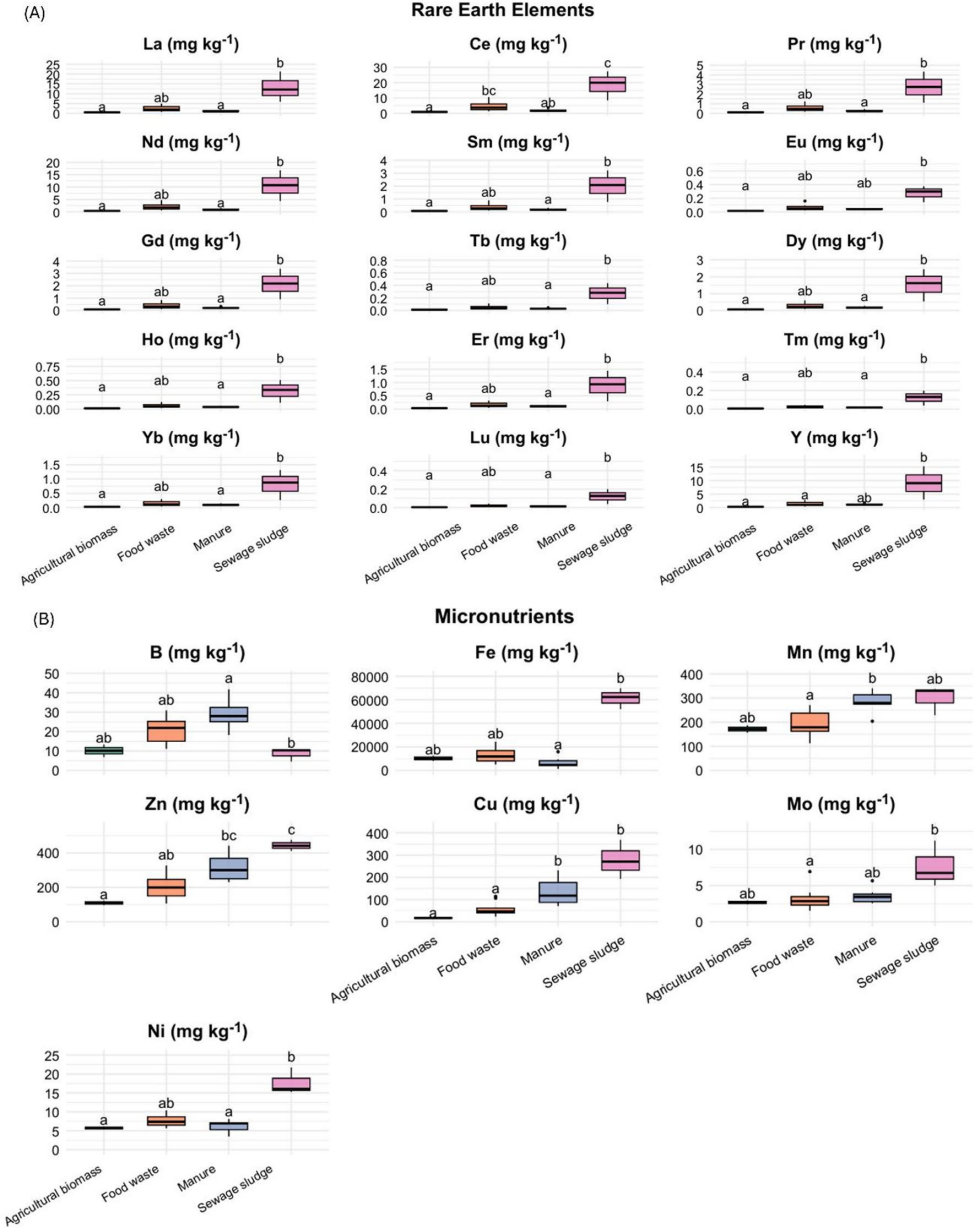
Concentration profiles of (**A**) rare earth elements and (**B**) micronutrients by feedstock origin. Boxplots show medians (center line) and interquartile ranges (IQR; boxes, Q1–Q3); whiskers extend to 1.5×IQR, and points represent individual observations; agricultural biomass (*n* = 2, green), food waste (*n* = 11, orange), manure (*n* = 7, purple), and sewage sludge (*n* = 3, pink). Lowercase letters indicate difference among feedstocks (Kruskal-Wallis test with Dunn’s test; Bonferroni-adjusted α = 0.05).

Elastic net (EN) and generalized additive models (GAMs) identified TAN, potassium (K), and boron (B) as the three most influential drivers of phytotoxicity (Figs. 2 and 3). Across 51 chemical variables, EN explained ~ 70% of GI variance (R² = 0.70; RMSE = 14.8 GI units), with strong concordance to false discovery rate (FDR)-controlled Spearman correlations and GAMs (Fig. 2; Supplementary Table 2).

Our models established a clear non-linear dose-response relationship, where increasing concentrations of TAN, K, and B sharply decreased GI. From these patterns, we determined performance-based cut-points of TAN ≥ 1,122 mg N L^− 1^, K ≥ 39.6 × 10^3^ mg kg^− 1^, and B ≥ 22.5 mg kg^− 1^ of dry matter, (DM). These thresholds informed a classification model that predicted phytotoxicity with high accuracy (sensitivity: TAN: 60%; K and B: 75%; specificity: TAN and B: 82%; and K: 91%), yielding an area under the curve (AUC) of 0.71–0.80). In contrast, iron (Fe) and several rare earth elements (REEs; e.g., La, Nd, Gd, Tb, Dy, Sm, Lu, Er, Ho, Yb and Tm) showed positive associations with GI within the observed ranges, suggesting potential stimulatory effects.

Overall digestate composition was consistent with these patterns (Supplementary Tables 3–4). Manure digestates combined high TAN (1,117.3 ± 294.9 mg N L^− 1^) with elevated K (48,866.74 ± 15,990.4 mg kg^− 1^ DM) and B (28.98 ± 7.51 mg kg^− 1^ DM). Food waste digestates paired high TAN (1,088.3 ± 406.9 mg L^− 1^) with raised K (33,176.97 ± 9,511.74 mg kg^− 1^ DM). In contrast, agricultural biomass and sewage sludge digestates showed lower TAN (< 1,017 mg L^− 1^), aligning with the higher GI in their liquid fractions (Supplementary Table 3).

### Micronutrients, rare Earth elements, and regulated metals

**Fig. 4:**
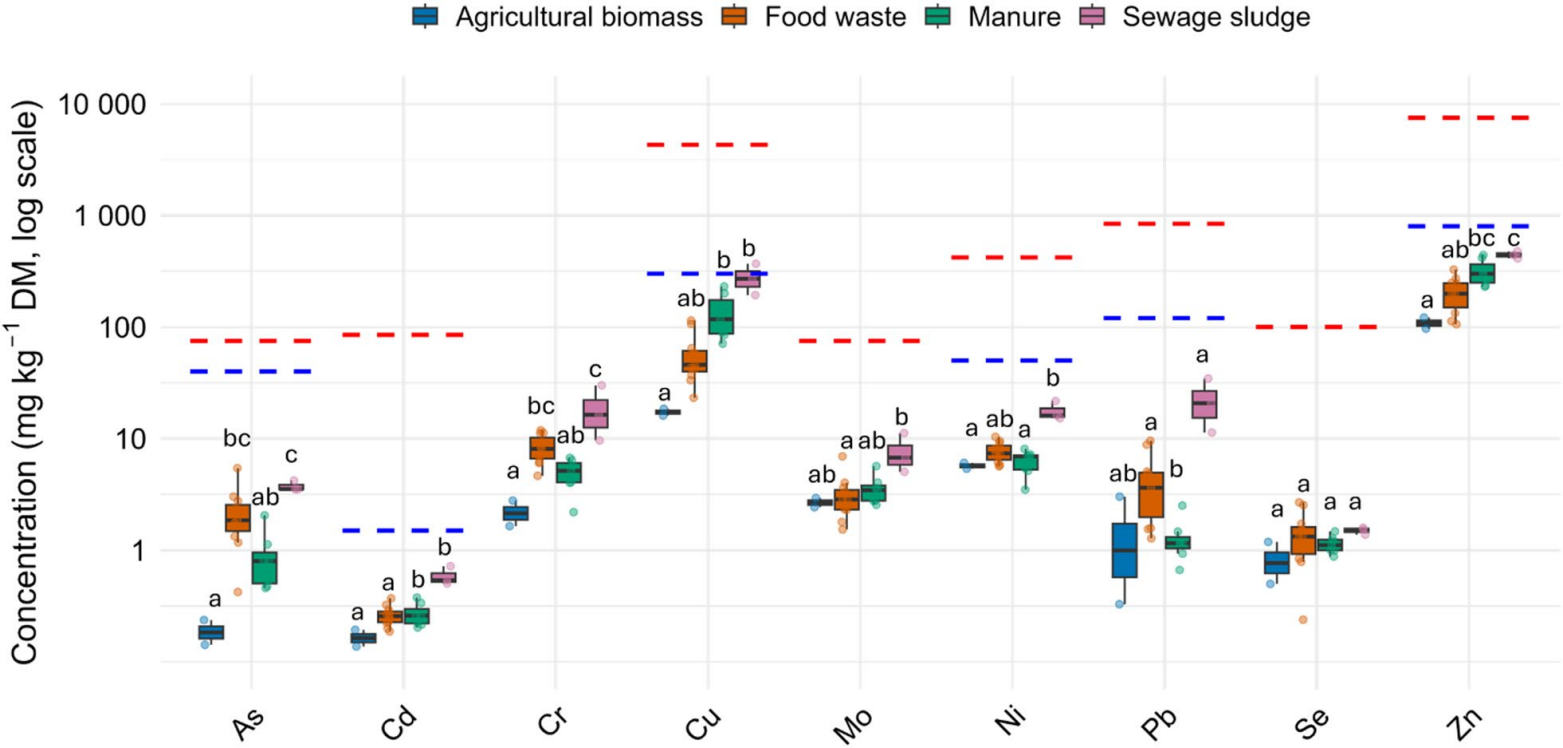
Heavy metals by feedstock and regulatory thresholds. Boxplots on a logarithmic scale (mg kg^− 1^ DM) show medians (center line) and interquartile ranges (IQR; boxes, Q1–Q3); whiskers extend to 1.5×IQR, and points represent individual observations; agricultural biomass (*n* = 2, blue), food waste (*n* = 11, orange), manure (*n* = 7, green), and sewage sludge (*n* = 3, pink). Regulatory thresholds from the European Union Regulation 2019/1009 (blue dashed lines) and the US EPA (40 CFR Part 503) (red dashed lines) are shown for comparison. No threshold for total Cr, only Cr(VI) regulated in EU (2 mg kg^− 1^ DM). Lowercase letters indicate difference among feedstocks (Kruskal-Wallis test with Dunn’s test; Bonferroni-adjusted α = 0.05).

Digestates contained a wide range of micronutrients, REEs, and heavy metals, with distributions strongly dependent on feedstock origin (Figs. 3 and 4). REEs were consistently enriched in sewage sludge digestates, particularly La (12.12 mg kg^− 1^ DM), Gd (2.2 mg kg^− 1^ DM), and Dy (1.61 mg kg^− 1^ DM; Fig. 3A). Among micronutrients, Fe was most abundant in sewage sludge digestates (Fe > 60,000 mg kg^− 1^ DM) and showed a positive correlation with GI (Fig. 2). Mn and Mo were also enriched in sewage sludge (330.3 and 6.74 mg kg^− 1^ DM, respectively), whereas B was elevated in manure and food waste digestates (M: 27.97 mg kg^− 1^ and FW: 21.9 mg kg^− 1^ DM), often exceeding the agronomic optimum of ~ 2 mg kg^− 1^ DM for crops^32^.

All regulated heavy metals (except Hg) were quantified, and all digestates concentrations complied with EU Regulation (2019/1009) for CE-marked PFCs (where applicable) and below US EPA 40 CFR 503 limits. Concentrations were highest in sewage sludge and lowest in agricultural biomass digestates, however all remained below regulatory thresholds.

Zn and Cu, although essential micronutrients, must be assessed from both nutrient and contaminant perspectives. They are regulated as contaminants in EU 2019/1009 product function category (PFC) 1(A) organic fertilizers (Cu ≤ 300 mg kg^− 1^; Zn ≤ 800 mg kg^− 1^ DM), and are also capped in PFC 1(B) organo-mineral products (Cu ≤ 600 mg kg^− 1^; Zn ≤ 1,500 mg kg^− 1^ DM), except when intentionally added and declared as micronutrients in accordance with Annex III^20^. In the US, both are regulated under EPA 40 CFR 503 with higher ceilings (Cu = 4,300 mg kg^− 1^; Zn = 7,500 mg kg^− 1^ DM). In our dataset, Zn and Cu mostly fell well below these limits, except for one sewage sludge observation. For agronomic context, Zn is typically beneficial in plant tissues at ~ 20–100 mg kg^− 1^ DM and may become phytotoxic above ~ 300 mg kg^− 1^ ^33^.

### TAN-K-B quantification as a performance-based framework for safe digestate circularity

**Fig. 5:**
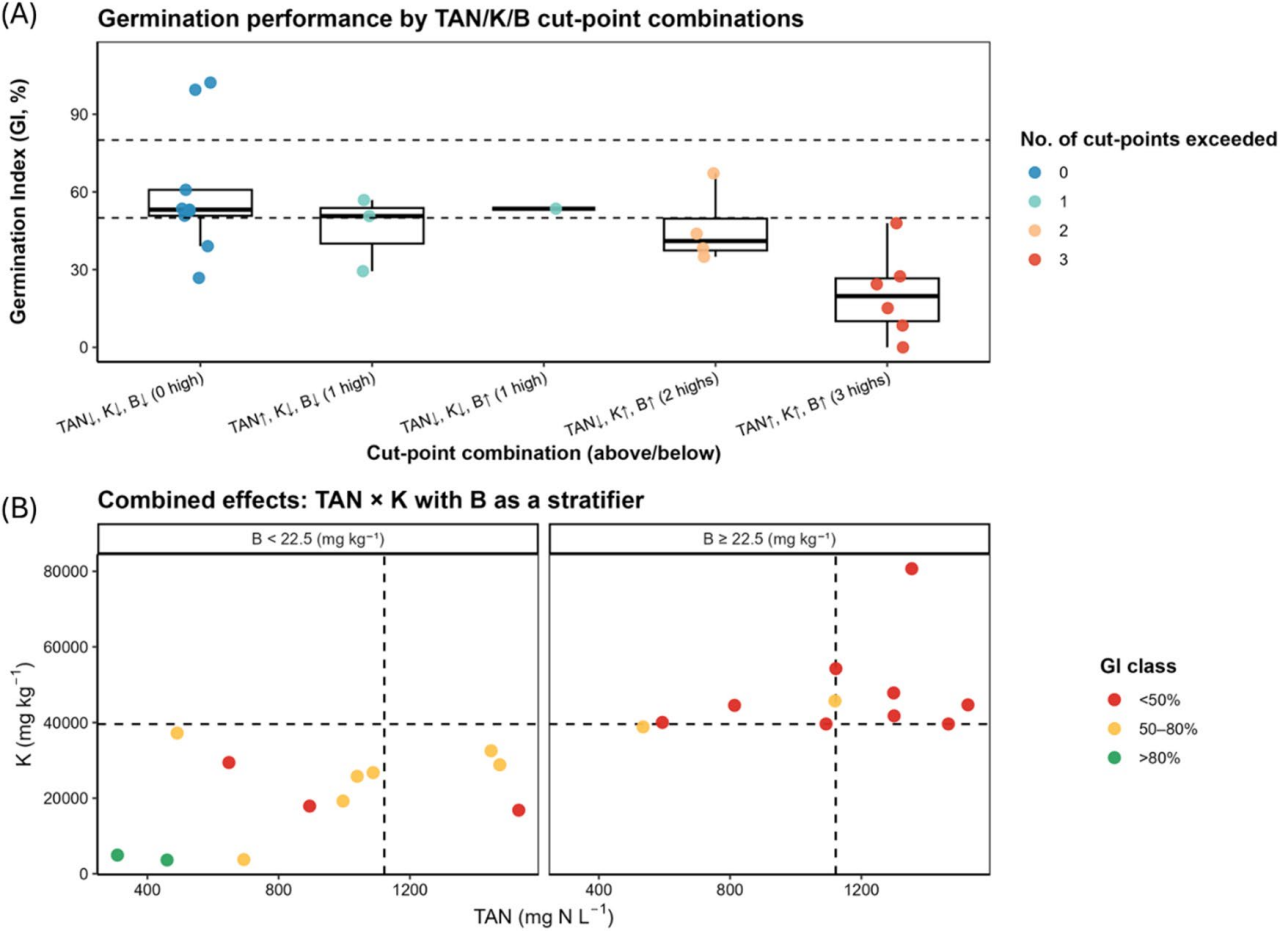
Combined effects of total ammonia nitrogen (TAN), potassium (K), and boron (B) on germination index (GI). (**A**) GI by cut-point combinations for TAN, K and B. Categories indicate whether each driver is below (↓) or above (↑) its cut-point (TAN = 1,122 mg N L^− 1^; K = 39,600 mg kg^− 1^; B = 22.5 mg kg^− 1^). Boxes show median and IQR; points are individual digestates samples. Dashed lines mark GI thresholds at 80% and 50%. Point color encodes the number of cut-points exceeded (0–3). (**B**) TAN–K scatterplots stratified by B (< 22.5 mg kg^− 1^ ≥ 22.5 mg kg^− 1^). Dashed lines indicate TAN and K cut points. Point color denotes GI class (> 80% non-phytotoxic; 50–80% moderately phytotoxic; < 50% highly phytotoxic).

**Fig. 6:**
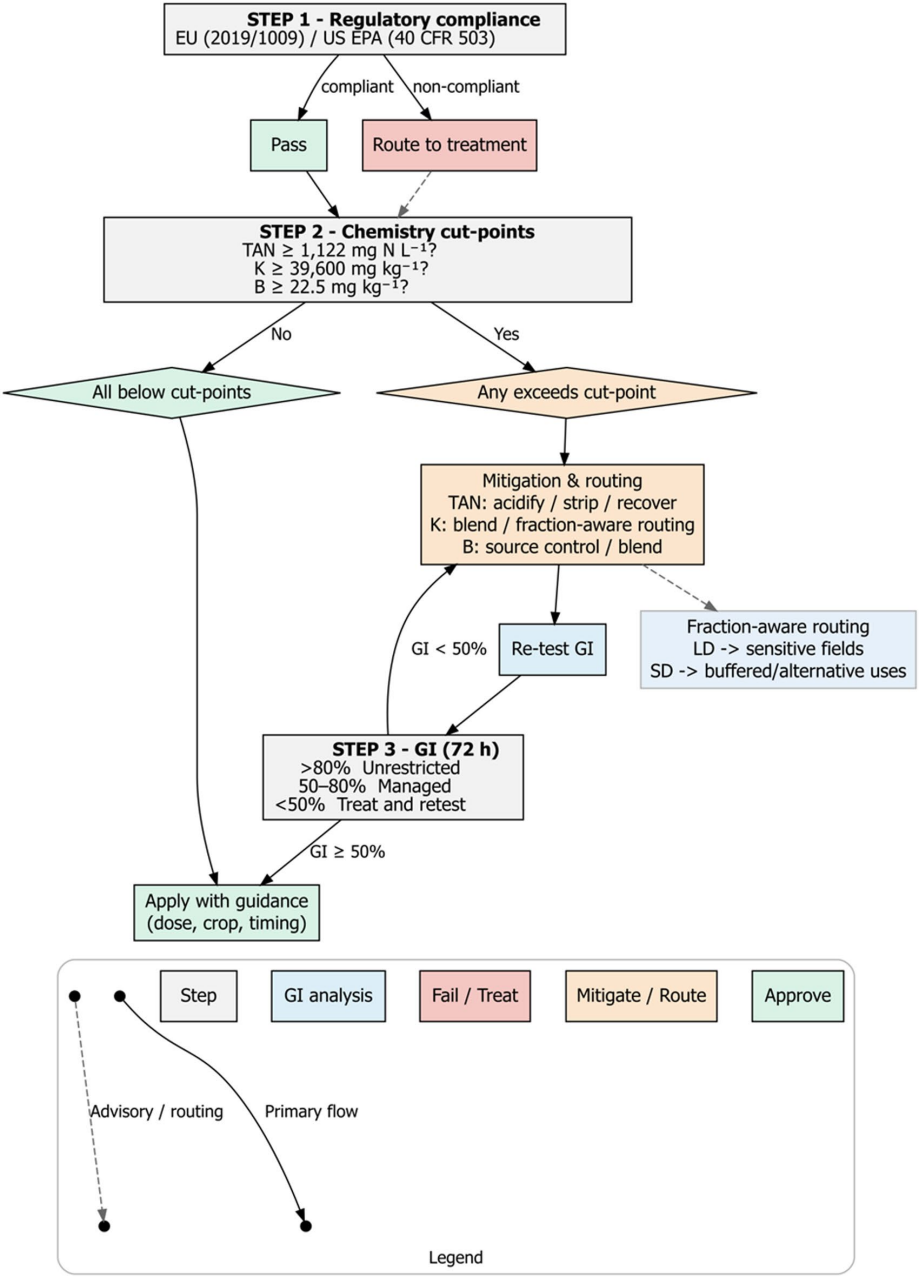
Performance-based decision flow for digestate reuse in agriculture. Flow diagram summarizing sequential screening elements (regulatory compliance, bioassay classification, chemical mitigation at TAN-K-B cut-point, fraction-based routing to field or alternative valorization).

Integrating GI bioassays with chemical thresholds allows routine screening to be reduced from dozens of analytes to three process-informed levers: TAN, K and B, which together explained most GI variance while keeping monitoring cost-effective (Figs. 5 and 6).

GI was generally > 50% when all three variables were below thresholds, whereas joint exceedance of TAN, K and B yielded uniformly high phytotoxic (GI < 50%). Mapping TAN versus K with B as a stratifier further underscored B as a critical modifier (Fig. 5B). Under low-B conditions (< 22.5 mg kg^− 1^), TAN and K effects appear more diffuse, but once B exceeded its thresholds, even moderate TAN and K levels often drove GI < 50%.

From these results, we propose a decision flow (Fig. 6). In the first step, digestates must comply with baseline regulatory ceilings (EU 2019/1009; US EPA 40 CFR 503). Non-compliant batches are routed to treatment or restricted to non-food uses, while compliant ones proceed to chemistry cut points. Batches exceeding thresholds for TAN-K-B are subjected to mitigation tailored to each element: acidification or stripping/recovery for TAN, blending or fraction-aware routing for K, and source control or blending for B. Mitigated digestates are then re-tested through a 72 h GI, which classifies digestates as unrestricted (GI > 80%), deployable with management (50–80%) or requiring retreatment (< 50%). Only batches that maintain GI ≥ 50% after mitigation are approved for agricultural use, applied under guidance on dose, crop and timing.

## Discussion

Our results show that digestate performance cannot be inferred from feedstock origin alone; neither sewage sludge nor manure are inherently safe or unsafe. The observed variability indicates that composition and fraction, rather than origin, govern germination responses across full-scale systems. The consistently high GI medians exhibited by sewage sludge digestates are counter-intuitive but attributable to a favorable chemical composition, specifically their lower concentrations of TAN-K-B, and enrichment in phytostimulatory elements (e.g., Fe, REEs).

Multivariate modelling identified TAN, K and B as independent drivers explaining ~ 70% of GI variance. TAN, defined as the sum of NH_4_^+^ and free NH_3_^34^, exerts toxicity via pH-dependent NH_3_ diffusion during imbibition, disrupting cytoplasmic homeostasis and inhibiting root elongation^7,23,35,36^. Excess K imposes osmotic and salinity stress, which limit water uptake and early metabolism and depresses P uptake^37,38^. B has a small safety margin between beneficial and toxic concentrations; even moderate elevations can induce oxidative stress and reduce cell elongation in sensitive species such as tomato^32,39^. Crucially, when all three drivers exceeded their cut-points, all digestates were highly phytotoxic (GI < 50%), supporting integrated thresholds rather than single-parameter monitoring.

Previous studies have usually assessed digestate-related plant risks using single parameters, most commonly TAN, pH or bulk salinity indicators such as electrical conductivity (EC)^7,22,24^. Excessive salt concentrations (EC: 4–8 dSm^− 1^ moderate; 8–16 dSm^− 1^ strong; >16 dSm^− 1^ extreme; sodium adsorption ratio: 13–18 medium; 18–26 high; >26 very high) are major causes of reduced agricultural productivity worldwide^40^. However, salinity metrics cannot indicate which ions drive inhibition since EC is shaped by the associated chemical and biochemical conditions, including nitrate, Mg^2+^ and Ca^2+^, and urease activity, rather than reflecting a single mechanistic driver^3^. Controlled experiments further show that plant responses arise from interactions among multiple ions: combined boron toxicity and salinity alter membrane stability, ion mobility, water transport and pH, producing physiological outcomes distinct from those of individual factors^19^. These limitations indicate why single-parameter metrics fail to explain germination responses. In contrast, a multi-parameter framework, such as the proposed TAN-K-B, offers chemically explicit thresholds that detect biologically unsafe digestates even when EC appears acceptable, while guiding mitigation directly toward specific inhibitory factors.

In addition to these dominant factors, secondary associations included positive correlations of Fe and selected REEs (e.g., La, Nd, Dy) with GI within the observed ranges, consistent with their roles in chlorophyll biosynthesis, redox balance and micronutrient stimulation reported in seed-germination assays^41^. For instance, La^3+^ at 0.05–1.5 mg L^− 1^ and Dy^3+^ at 0.09 mg L^− 1^ have been shown to stimulate germination and root elongation in several crops^30,42,43^. These patterns are mechanistically consistent with plant physiology. Fe is central to chlorophyll biosynthesis, Fe–S cluster formation and photosynthetic electron transport, and moderate increases in its availability can temporarily enhance redox balance and chloroplast function before excessive Fe catalysis Fenton-type reactions leading to reactive oxidative stress (ROS) formation^41^. Thus, the stimulatory correlations observed for Fe and REEs fall within the expected window where micronutrient supply supports metabolic activation rather than oxidative stress^41^. However, these effects were highly dose- and species-dependent and often reversed at higher concentrations, so they should be regarded as contextual rather than operational until validated by long-term dose-response and speciation studies.

Translating these mechanistic findings into management practice, our results provide actionable levels for operators. Acidification, stripping/recovery or soil injection may be used to mitigate phytotoxicity by lowering TAN while curbing NH_3_/N_2_O emissions. Blending streams or adjusting process parameters can reduce K-driven ionic strength, while B hotspots can be managed through source control or dilution. Solid-liquid separation remains a powerful management tool. Liquids concentrate plant-available N and K, while solids retain P, organic matter and trace metals, enabling targeted routing and compliance^11,29^. On average, 65–75% of total N (mostly ammonium-N) and 70–80% of K remain in LD, whereas 55–65% of P and 60–70% of C remain in SD^29^. This redistribution allows targeted routing: liquids to fields requiring immediate nutrient uptake, and solids to buffered soils or alternative valorization pathways such as pyrolysis or hydrothermal carbonization.

The technological choices for this separation range from conventional mechanical options – such as screw presses, decanter centrifuges, belt presses, rotary drums and vibrating screens – to advanced solutions including membrane filtration, ammonia stripping and scrubbing, or thermal processes. These technologies influence not only logistics and nutrient recovery but also the biological safety of digestate^11^. By reducing handling volumes and enabling tailored nutrient application, solid-liquid separation acts as a dual lever: mitigating acute phytotoxicity by managing the liquid fraction, where TAN and K are concentrated, and reducing long-term metal accumulation by directing the solids fraction to appropriate management routes. These practices are already promoted under EU instruments such as the Nitrates Directive 91/676/EEC and support both agronomic performance and regulatory compliance^11,44^.

Beyond these conventional levers, emerging electrochemical processes further expand the mitigation toolbox when TAN exceeds cut points. Laboratory trials show that potassium nickel hexacyanoferrate (KNiHCF)-based system can recover ~ 68% of NH_4_^+^ from real manure wastewater with high selectivity (~ 93–98% over competing cations), while simultaneously co-producing hydrogen (H_2_) or hydrogen peroxide (H_2_O_2_) with faradaic efficiencies above 80%^45^. This integrated approach not only mitigates ammonia-related phytotoxicity but also enables valorization pathways by generating fertilizers and value-added chemicals, thereby linking digestate management to broader goals of nutrient recovery, pollution control, and decentralized bioeconomy solutions^45^.

By translating mechanistic insights into three measurable drivers (TAN, K, and B) and verifying them with GI bioassays, we propose a decision-ready framework for digestate governance. Moving beyond feedstock-based restrictions, this strategy enables outcome-oriented evaluation, allowing safe digestates to circulate irrespective of origin and embedding transparent, verifiable standards in certification schemes or procedure contracts, reducing farmer concerns and expand market opportunities.

The need for outcome-based assurance is underscored by the current N policy landscape. A global database identifies 2,726 N-related policies across 186 countries, with only 28 integrating across multiple sinks, conditions that heighten risks of pollution swapping^46^. Our TAN-K-B screen, tied to operational levers, provides a cross-sink criterion that helps avoid such trade-offs and supports integrated targets. Agriculture drives 9–14% of global GHG emissions (excluding land-use change), ~ 70% of freshwater withdrawals and ~ 78% of eutrophication, so routing digestate via performance-based thresholds directly targets impacts hotspot^47^. Because TAN management simultaneously reduces NH_3_ volatilization and N_2_O formation while safeguarding crop establishment, the rule connects agronomic performance with air-quality and climate objective^14,29^. Together, TAN, K and B define a practical index that integrates biological safety, nutrient-use efficiency (NUE) and environmental integrity, establishing a functional safe operating space for agricultural nutrient flows^1^.

Nevertheless, several limitations and research need remain. Our GI bioassays (72 h) capture accurate responses but not long-term soil-plant-microbiome dynamics or multi-season effects^29^. The models focused on chemical parameters; emerging contaminants such as persistent herbicides, antibiotics, pharmaceuticals and microplastics, were not measured and may contribute to unexplained variability. For example, herbicides such as aminopyralid and picloram, common in livestock feed-manure pathways, may hinder seed germination and thus contribute to the reduced GI observed in our assays^26–28,48^. Associations of Fe and REEs with higher GI are intriguing but require caution; effects are highly dose- and species-dependent and should not be operationalized without long-term dose–response and speciation studies^30,42^.

Future research should pair digestate screening with field- and regional-scale management indicators, most notably N surplus and NUE. Evidence shows that improving NUE is the most powerful mechanism to reduce fertilizer-related GHGs^49^. However, sub-regional heterogeneity – for example, mean savings of ~ 18 kg N ha^− 1^ and ~ 32% NUE gains in South Asian rice systems – demanding locally calibrated targets^49^. Instead of simply embedding outcome-based digestate criteria in integrated policy roadmaps, future work should test how these criteria can be operationalized within existing governance frameworks, evaluate trade-offs across sinks and sectors, and identify institutional conditions that enable adoption at scale^46^. To consolidate these advances, multi-scale assessments are needed, including (i) greenhouse and field trials to capture chronic and cumulative effects, (ii) life-cycle analyses linking digestate routes to GHG emissions and planetary boundaries for N, P and metals, and (iii) expanded screening for trace organics and micropollutants. Together, these efforts will provide a comprehensive risk-benefit framework, ensuring that digestate valorization strengthens food security, soil health and climate mitigation.

## Conclusion

This study demonstrates that digestate performance and safety are governed by composition and fraction rather than feedstock origin. By integrating germination bioassays with operational cut-points for TAN, K and B, we establish a performance-based framework that reliably distinguishes low- from high-risk products across 23 full-scale digestates. This approach identified safe fractions for agricultural use and flagged batches requiring mitigation before field application. Although all samples complied with EU Regulation 2019/1009 for CE-marked PFCs (where applicable) and US EPA 40 CFR 503 limits, sewage sludge and manure digestates occasionally approached Cu and Zn thresholds, emphasizing the importance of cumulative load management. By shifting from origin-based restrictions to performance-based assessment, our framework enables flexible, site-specific routing of digestates, maximizing nutrient recovery while safeguarding crop establishment and soil integrity. Because agriculture is a dominant driver of GHG emissions, freshwater withdrawals and eutrophication, embedding these criteria into regulatory and certification systems would not only accelerate the safe expansion of digestate reuse but also strengthen farmer confidence, support integrated nitrogen governance across sinks, and advance nutrient circularity. Framing digestate use through outcome-based rules therefore offers a scalable pathway to close nutrient loops and enhance food system resilience without breaching planetary boundaries.

## Methods

### Digestate sampling

Digestate samples were collected from the main primary digesters of 23 full-scale biogas plants across Sweden, Norway, and Denmark. The sampling campaign took place between August and December 2022, as part of a coordinated effort by the Biogas Solutions Research Center (Linköping, Sweden). Digesters were classified by dominant feedstocks as agricultural biomass (AB, *n* = 2), food waste (FW, *n* = 11), manure (M, *n* = 7), and sewage sludge (SS, *n* = 3), and included both mono-digestion and co-digestion systems with co-feedstocks from municipal, agricultural and industrial sources (Supplementary Table 5).

Operating conditions varied across sites, with mesophilic and thermophilic conditions (36–56 °C). Reported hydraulic retention times (HRT) ranged from 20 to 43 days and organic loading rates (OLR) from 2.0 to 5.4 kg VS m^− 3^ d^− 1^ (Supplementary Table 4). The sample digesters also differed in hygienization and pre/post-AD treatments, including thermal sanitization, and chemical pre-treatment.

For each digester, triplicate samples (10 L each) were collected from effluent ports. Samples were transported to the laboratory and kept in a water bath at their respective operating temperatures within the first 24 h after arrival to preserve microbial and chemical integrity. During this period, subsamples were taken from each digestate for chemical analyses.

### Digestate characterization

All analyses were performed on each of the three replicate samples collected per plant. pH was measured within 24 h after arrival at the laboratory using a pH meter (InoLab 7310, WTW, Germany). Total solids (TS) and volatile solids (VS) were determined gravimetrically, following standard protocols^50^. Approximately 15 g of homogenized digestate was dried at 105 °C for 24 h to determine TS, and the residue was then ignited at 550 °C for 2 h in a muffle furnace (Nabertherm, Germany) to determine VS.

Volatile fatty acids (VFAs, namely: acetate, propionate, butyrate, isobutyrate, valerate, isovalerate, caproate, isocaproate) were analyzed by gas chromatography (8860 GC System, Agilent, USA). For each sample, 2 mL of digestate was centrifuged at 12,000 rpm for 10 min, and 400 µL of supernatant was transferred to glass vials with 40 µL of crotonic acid as internal standard.

Total ammonia nitrogen (TAN) was determined after centrifugation (10,000 rpm, 10 min) and filtration through 0.45 μm polyethersulfone (PES) syringe filters (VWR International, USA). Filtrates were stored at − 20 °C, thawed, and diluted up to 7,900 times before analysis using an AutoAnalyzer (SEAL Analytical, USA). Free ammonia nitrogen was calculated as described previously^34^:

1 $$\:{NH}_{3}-N\:=\frac{TAN}{1+\frac{{10}^{-pH}}{\:{10}^{-\left(\mathrm{0,09018}+\frac{\mathrm{2729,92}}{T}\right)}}}$$

Total organic carbon (TOC) and total organic nitrogen (TON) were determined in dried (60 °C) and milled samples using a CHN elemental analyzer (Thermo Fisher, Flash 2000).

Trace elements and heavy metals were measured by inductively coupled plasma mass spectrometry (ICP-MS, Agilent 8900). Reaction/collision gases were selected according to the element to minimize interferences. No gas was used for Li, Be, and B; helium was used for Na, Mg, Al, K, Ca, Ti, V, Cr, Mn, Fe, Co, Ni, Cu, Zn, Sr, Mo, Ag, Cd, Sb, Ba, Tl, Pb, and Bi; and O₂ was used for As and Se.

### Phytotoxicity bioassays

Phytotoxicity was evaluated in vitro using commercial tomato seeds (*Solanum lycopersicum*), a species widely recognized for its high sensitivity to herbicides and environmental contaminants^25,26^. Bioassays followed a published protocol^31^.

Three digestate fractions were tested: WD, LD, and SD, each diluted 1:10 (w/v) with deionized water. LD and SD were obtained by centrifuging WD at 10,000 rpm for 10 min at 20 °C (Beckman Coulter Avanti J-E). Digestate centrifugation reflects a common post-treatment practice in full-scale digestate management to facilitate handling and targeted nutrient applications.

Phytotoxicity tests with WD were prepared by mixing 1 g of homogenized digestate with 9 mL of deionized water. For LD experiments, 1 g of the clarified supernatant with 9 mL of deionized water. The centrifuged portion of the digestate was dried at 70 °C for 20 h to obtain SD, 1 g of which was them mixed with 9 mL of deionized water. The use of the same wet weight (1 g) for all fractions, rather than standardizing by TS, VS, or nutrient concentration, was intentional to compare phytotoxic responses across the natural range of digestate properties. Such differences are considered part of the treatment effect in this experimental design.

The prepared solutions (10 mL) were used to incubate 10 seeds per Petri dish, at room temperature (24 ± 1 °C) for 72 h. As a control, seeds were incubated with 10 mL of deionized water. All incubations were performed in triplicate.

The GI was calculated according to Eq. 2:

2 $$\:\mathrm{G}\mathrm{I}\:\left(\mathrm{\%}\right)=\frac{RSG\:\left(\mathrm{\%}\right)x\:RRG\:\left(\mathrm{\%}\right)}{100}$$

where RSG is the relative seed germination (Eq. 3), i.e., the percentage of seeds germinated in treatment compared with control, and RRG is the relative root growth (Eq. 4), i.e., the percentage of mean root length in the treatment compared with the control.

3 $$\:\mathrm{R}\mathrm{S}\mathrm{G}\:\left(\mathrm{\%}\right)=\frac{{N}_{SG,T}}{{N}_{SG,B}}\:\times\:100$$

4 $$\:\mathrm{R}\mathrm{R}\mathrm{G}\:\left(\mathrm{\%}\right)=\frac{{L}_{R,T}}{{L}_{R,B}}\:\times\:100$$

where N_SG, T_ and N_SG, B_ are the mean numbers of germinated seeds in the treatment and control, respectively, and L_R, T_ and L_R, B_ are the corresponding mean root lengths. Root lengths were measured manually using a digital caliper to the nearest 0.01 mm. GI values below 50% were interpreted as indicative of phytotoxicity^23^.

### Statistical analysis

All analyses were conducted in R v4.2.3. The normality of all variables was tested using the Shapiro-Wilk test. As most variables deviated from normality (*p* < 0.05), non-parametric analyses were applied. Differences between feedstocks and organic fractions were evaluated using the Kruskal-Wallis test, followed by Dunn’s post hoc test with Bonferroni correction.

To identify chemical drivers of phytotoxicity or phytostimulation, the GI was correlated with 51 chemical parameters using three approaches: (i) Spearman’s rank correlation with Benjamini-Hochberg false discovery rate control, reporting correlation coefficients (ρ), two-sided p-values, and FDR-adjusted q-values; (ii) EN regression with α = 0.5 and predictors standardized (z-scores), where the penalty parameter λ was chosen via 10-fold cross-validation using the 1-SE rule (λ₁se) and results are presented as standardized coefficients (β) and model performance metrics (RMSE, R²); and (iii) GAMs fitted for GI as a function of each candidate predictor using thin-plate regression splines (REML, k ≤ 4), with p-values for the smooth term and the estimated degrees of freedom reported.

A predictor was retained as a robust driver if it met the following criteria: (i) significant in Spearman correlation (q < 0.05) or non-zero β in EN, and (ii) significant in GAM (*p* < 0.05), with the same effect direction (positive/negative association) across all methods. For each retained driver, we derived univariate cut-points to classify GI < 50% (indicative of phytotoxicity) using receiver operating characteristic (ROC) analysis (pROC). Cut points were chosen to maximize Youden’s J and are reported with their associated sensitivity, specificity, and AUC. All details on statistical procedures, data preprocessing, and plotting parameters are provided in Supplementary Methods S1.

## Supplementary Information

Below is the link to the electronic supplementary material.

Supplementary Material 1

Supplementary Material 2

## Data Availability

All data underlying the figures and analyses are provided as Source Data files. Additional processed tables and metadata are available in the Supplementary Material.
