# Supplementary material for: Performance-based criteria for safe and circular digestate use in agriculture
Source: Sci Rep. 2025 Dec 24;16:3369. doi: 10.1038/s41598-025-33314-x (PMC12834953; doi:10.1038/s41598-025-33314-x)
Supplement: Supplementary file 1 — Supplementary Material 1 [file 41598_2025_33314_MOESM1_ESM.docx]

**Performance-based criteria for safe and circular digestate use in agriculture**

# **SUPPLEMENTARY MATERIAL**


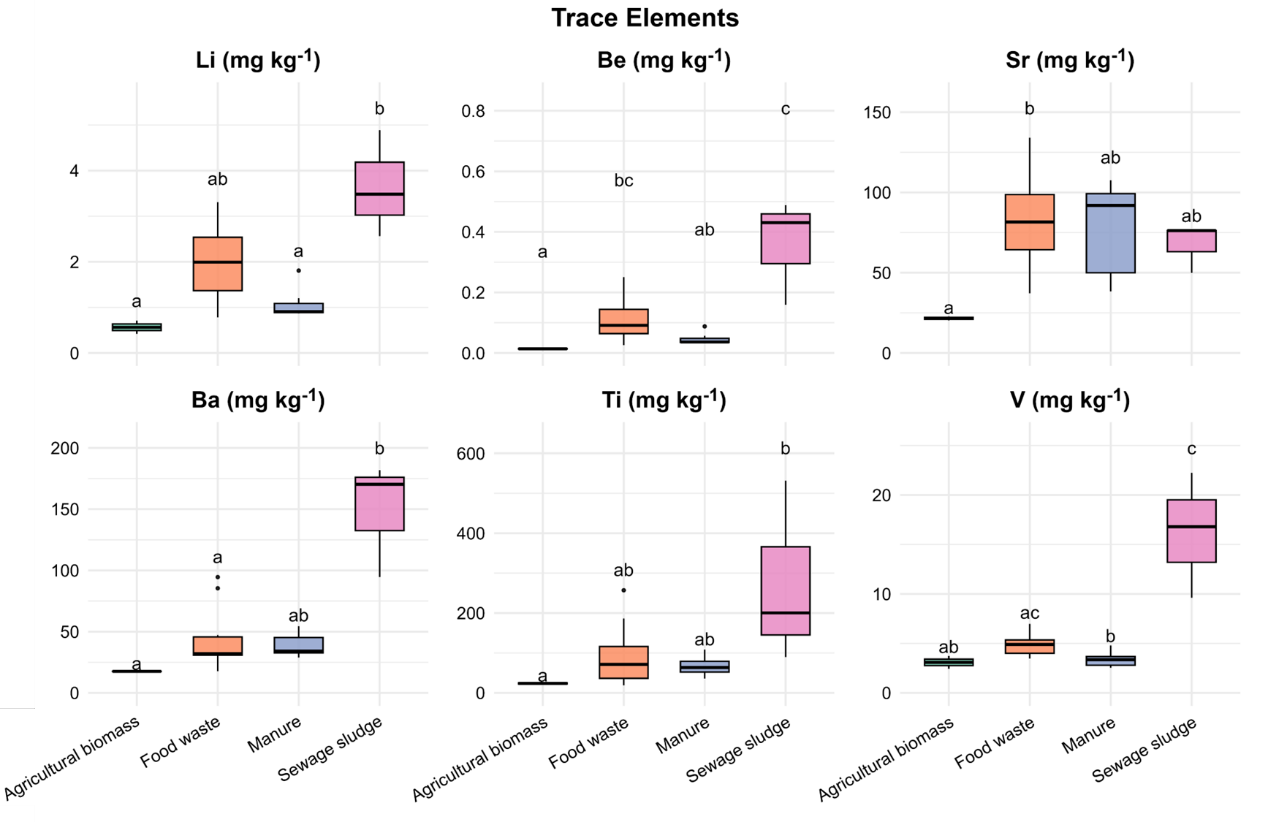


**Supplementary Fig. 1. Concentration profiles of trace elements in digestates from different feedstock origins.** Boxplot represents agricultural biomass (n = 2, green), food waste (n = 11, orange), manure (n = 7, purple), and sewage sludge (n = 3, pink). Statistical differences were assessed using Kruskal’s Wallis and Dunn test (p < 0.05).

**Supplementary Methods – Statistical analysis**

All analyses were performed in R (v4.2–4.3) R with scripts archived in the “Digestate” GitHub repository (see Code availability). Normality was evaluated using the Shapiro–Wilk test (shapiro.test). Because many outcomes were bounded (e.g., GI: 0–100%) and groups were unbalanced, normality was frequently rejected (p < 0.05) or visually uncertain, and non-parametric tests were applied to all group comparisons.

Differences across categories (e.g., feedstock within fractions, fractions overall) were tested with Kruskal–Wallis followed by Dunn’s post-hoc comparisons with Bonferroni correction. Results are shown as boxplots with raw data overlay and compact letter displays for groupings (Figs. 1, 3, 4, S1).

Associations between GI and chemical predictors were quantified by Spearman’s rank correlation with FDR adjustment. Variables with FDR < 0.05 or non-zero Elastic Net coefficients (glmnet, α=0.5, λ via 10-fold CV) were retained for GAM testing (mgcv, thin-plate splines). A variable was considered a GI driver if it passed both screening and GAM significance. Robustness was summarized with lollipop plots, correlation–effect scatterplots, and a heatmap integrating the three methods (Fig. 2).

Rare earth elements and micronutrients were profiled by feedstock category (Figs. 3A–B), while heavy metals were compared with EU and US EPA thresholds (Fig. 4). Cut points for TAN, K, B, and Fe were derived via ROC analysis (pROC) using Youden’s J index; sensitivity, specificity, and AUC are reported in Table S1. GI outcomes relative to these thresholds are shown as boxplots and TAN–K interactions stratified by B (Figs. 5A–B).

Finally, a decision flowchart (Fig. 6) was generated (DiagrammeR, DiagrammeRsvg, rsvg) to illustrate potential screening strategies. Figures were produced with ggplot2 and companion packages (ggrepel, ggtext, patchwork, scales), exported as TIFF (300 dpi).
